# New Periodical, *Buffalo Medical and Surgical Journal and Reporter*

**Published:** 1861-09

**Authors:** 


New Periodical.—We have received the first number of the *Buffalo Medical and Surgical Journal and Reporter*, edited by Julius F. Miner, M. D., Surgeon to Buffalo General Hospital. Its editor "desires to say that he is looking to the profession for the means of success in the undertaking, and will not allow himself alone to be held responsible for its success or failure, since he is depending not so much upon his own exertions as the assistance he expects to receive from others. He enters upon the duty stimulated by the hope and expectation of so complete success, as soon to justify the enlargement of the *Journal*, rendering it more acceptable and useful than it can possibly become in its present restricted limits."

We welcome the *Journal and Reporter* to our exchange list, and trust its "hope and expectation" may be fully and speedily realized.

